# Feline Infectious Peritonitis: A Challenging Diagnostic and Therapeutic Labyrinth

**DOI:** 10.3390/ani16010128

**Published:** 2026-01-02

**Authors:** Violetta Iris Vasinioti, Maria Stella Lucente, Cristiana Catella, Canio Buonavoglia, Nicola Decaro, Annamaria Pratelli, Paolo Capozza

**Affiliations:** Department of Veterinary Medicine, University of Bari, 70010 Valenzano, Italy; violetta.vasinioti@uniba.it (V.I.V.); mariastella.lucente@uniba.it (M.S.L.); cristiana.catella@uniba.it (C.C.); nicola.decaro@uniba.it (N.D.); annamaria.pratelli@uniba.it (A.P.); paolo.capozza@uniba.it (P.C.)

**Keywords:** feline coronavirus, FIP, diagnosis, treatment options

## Abstract

Feline coronaviruses (FCoVs) are ubiquitous pathogens, exhibiting high prevalence across feline populations worldwide. Although the virulent mutated biotype feline infectious peritonitis virus (FIPV) is observed in only a small percentage of cats, it causes a systemic and often fatal disease. Diagnosis of feline infectious peritonitis (FIP) is challenging due to its non-specific clinical signs and the difficulty in differentiating between the two biotypes, feline enteric coronavirus (FECV) and FPIV. Currently, veterinarians rely on a combination of diagnostic methods, integrating laboratory tests, anamnesis and clinical signs to improve the diagnostic accuracy of FIP. Once considered untreatable, FIP now benefits from recent pharmacological advances that suggest promising therapeutic options, including antiviral drugs and immunomodulatory therapies. Despite these developments, the lack of an effective vaccine and definitive curative treatment highlights the need for continued research. This review provides a comprehensive analysis of the current literature on diagnostic and treatment approaches for FIP. The aim is to improve understanding of the available options and strategies for FIP to mitigate its severe consequences.

## Simple Summary

Feline infectious peritonitis (FIP) is a serious and often fatal disease in cats caused by a feline coronavirus. However, not all infected cats will develop FIP, as the virus must undergo a mutation to transform into the highly virulent form responsible for the disease. Identifying the cats infected with the mutated feline coronavirus remains a major diagnostic challenge, and without treatment, affected cats have limited chances of survival. This study provides an overview of the available diagnostic approaches and the treatment options that have recently emerged due to scientific advancements.

## 1. Introduction

Feline infectious peritonitis (FIP) was first described in 1966 [1], and since then, it has been reported in the feline population worldwide. It is a progressive systemic viral disease characterized by high mortality rates and remains one of the most challenging diseases in cats [2]. FIP typically infects young cats, particularly those under two years of age, and its transmission is favored in multi-cat environments. Although it primarily affects domestic cats, several cases have also been detected in wild felids [3,4,5].

The causative agent of FIP is the hypervirulent form of the feline coronavirus (FCoV), an enveloped single-stranded RNA virus of the family *Coronaviridae*, genus *Alphacoronavirus* 1 [6]. In detail, FCoV, based on its pathogenic potential, occurs in two biotypes: the most common and avirulent feline enteric coronavirus (FECV) and the highly virulent feline infectious peritonitis virus (FIPV) [7]. FECV is a highly contagious virus that can infect cats via the oral-fecal route targeting the gastrointestinal epithelium and is responsible for asymptomatic infection or mild to transient diarrhea. In contrast, FIPV is widely believed to arise from spontaneous mutations in FECV, which alter its cell tropism to monocytes/macrophages, thereby enabling FIP pathogenesis [8,9]. The exact mechanism of the mutation events responsible for FIP remains unclear [10], although previous studies have implicated the involvement of sequence variations in the spike (S) protein [11], membrane (M) protein [12], or the non-structural protein (NSP) 3c which is correlated with enteric tropism [9]. Two specific aminoacid substitutions in the S gene, previously thought to drive the shift in FCoV cell tropism [13], were subsequently associated with the systemic spread of FCoV rather than with the development of FIP per se [14]. Indeed, not all cats harboring the mutant FECV developed FIP [15]. In 2007, Dye and Siddell questioned the internal mutation theory by detecting identical 3c-mutated viruses in the gut and liver of a cat with FIP [8]. The theory of a 3c-mutated FIPV in the gut was later explained as a rare spillover from systemic lesions rather than a primary enteric infection [16]. As reported by Tekes and Thiel, FIPV replicates in the monocytes/macrophages, triggering their activation and, consequently, an increased production of cytokines (tumor necrosis factor-α, IL-1β) and adhesion molecules (CD11 and CD18). The interaction of monocytes with endothelial cells in the veins induces an enhanced vascular permeability, resulting in effusion [17].

FCoV is classified into two genotypes, FCoV type I and type II, which are distinguished by antigenic differences [18,19]. Interestingly, the two FCoV genotpes are not exclusive to a single disease outcome, being detected in both FECV and FIPV biotypes. Early studies proposed that FCoV type II originated through genetic recombination events between FCoV type I and canine coronavirus (CCoV), creating a chimeric version of FCoV with incorporation of the CCoV’s S gene [20,21]. Recently, Jaimes et al. suggested that FCoV type I and FCoV type II should be designated as separate viral species, emphasizing that structural and functional differences in the S proteins may play a decisive role in pathogenesis [22]. Despite its higher prevalence, FCoV type I is understudied due to its restricted replication in macrophages, which complicates isolation in cell culture [23]. In contrast, the epidemiologically less common FCoV type II can be easily propagated in cell culture and is known to use the feline aminopeptidase-N as a cell receptor for viral attachment and entry [24,25]. The cellular receptor of FCoV type I is still unknown, although according to Van Hamme et al., (2011) the feline dendritic cell-specific intercellular adhesion molecule grabbing nonintegrin (fDC-SIGN) is associated with FIPV type I infection of monocytes [26].

FIP can be presented with two distinct clinical manifestations, the effusive (wet) form and the non-effusive (dry) form [27]. Effusive FIP is characterized by pathological accumulation of fluid within body cavities, fibrinous pleuritis and peritonitis. Non-effusive FIP involves the development of granulomatous lesions in multiple organs, including ocular, pulmonary, and central nervous system (CNS) tissues [28]. Most of the FIP clinical cases are predominantly effusive, while some cases may exhibit a combination of both wet and dry forms.

Due to the potential of multi-organ involvement, the clinical signs and laboratory abnormalities of FIP can vary significantly. This variability makes accurate antemortem diagnosis very challenging, especially in the absence of effusion. Therefore, establishing reliable diagnosis pathway is crucial for the effective management of FIP cases, especially considering the promising results emerging from research on new therapies. This review summarizes current knowledge on diagnostic and treatment approaches for FIP. The aim is to improve understanding of available options and provide guidance to mitigate the severe consequences of this disease.

## 2. Ante-Mortem Diagnostic Approaches

**Figure 1:**
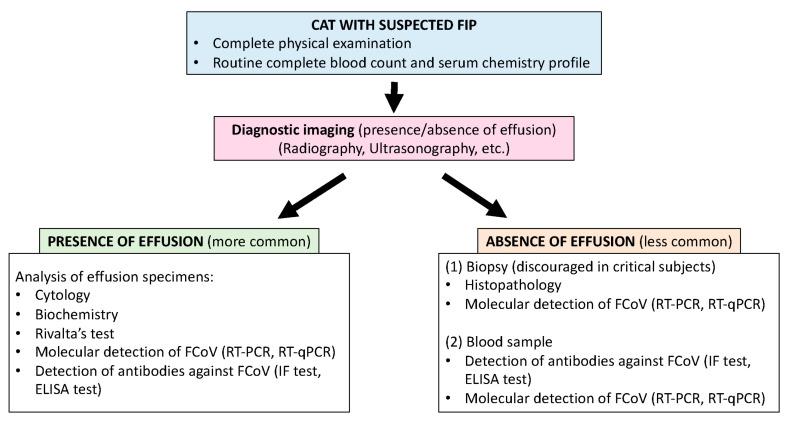
Workflow outlining the diagnostic tools commonly applied in the evaluation of cats suspected of having FIP.

To date, FIP cannot be diagnosed using a single, unequivocal diagnostic test, since the clinical signs and laboratory findings are not specific and can mimic different diseases. It is required to integrate multiple diagnostic tools (Figure 1) and to interpret the results with caution based on a personalized approach for each cat patient. Herein, the conventional diagnostic approaches that, when evaluated collectively, can confirm FIP development, are described. A summary of these approaches and their relative clinical utility is provided in Table 1.

### 2.1. Anamnesis and Physical Examination

Several risk factors associated with the development of FIP in cats should be considered during the history evaluation. For instance, young cats, particularly those younger than 2 years of age, are more susceptible to FIP [37]. Both sexes can develop the disease, although a slightly higher risk has been reported in male cats [38]. Furthermore, multi-cat environments facilitate the transmission of FCoV, and persistently high viral loads may increase the likelihood of FIPV emergence [39]. Studies have described a greater predisposition of purebred cats to FIP, while several stressors, including adoption, vaccinations and neutering, may contribute to the development of FIP in FCoV-infected cats [40,41].

Non-specific clinical signs of FIP that usually occur in the early stages of the disease include anorexia, lethargy, fever, lymphadenopathy and weight loss [28]. In the effusive form of FIP, the most common clinical presentation is the abdominal distension resulting from ascites [42]. Effusion can also occur in other body cavities like the pericardium and the pleural space [43,44,45]. Pleural effusion could be clinically suspected by the presence of dyspnea, tachypnea and attenuated heart sounds. Non-effusive FIP is more difficult to diagnose and is characterized by pyogranulomatous lesions and masses in internal organs. These lesions can produce different clinical signs depending on the organs involved, most commonly the kidneys, liver, gastrointestinal tract, eyes and nervous system [46,47,48]. Consequently, clinical examinations should be thorough and include ophthalmological, neurological, dermatological and comprehensive systemic evaluations. Although clinical signs are not pathognomonic for FIP diagnosis, they are valuable in guiding differential diagnosis and establishing clinical suspicion. Since clinical signs may evolve over time, repeated physical examinations are recommended. More detailed diagnostic and clinical guidelines for veterinary practitioners are provided by the European Advisory Board on Cat Diseases (ABCD) and the American Association of Feline Practitioners [2,29].

### 2.2. Routinary Laboratory Analysis

#### 2.2.1. Hematology

Hematological abnormalities in cats with FIP are generally nonspecific, although lymphopenia, microcytosis, anemia, band neutrophilia and thrombocytopenia are commonly observed [37,49]. While serum biochemistry provides a higher diagnostic value, it is not pathognomonic. Hyperproteinemia and particularly hyperglobulinemia are the most common serum finding in cats with FIP (observed in approximately 89%), and sometimes in combination with hypoalbuminemia and a reduced albumin-to-globulin (A:G) ratio, typically less than 0.4 [29]. Conversely, an A:G ratio greater than 0.8 makes FIP unlikely [42,50]. In some cases, and depending on the FIP type, hyperbilirubinemia in the absence of hemolysis and elevated liver enzymes may support suspicion of the disease [49]. Acute phase proteins (APPs) such as the α1-acid glycoprotein (AGP) and serum amyloid A are commonly elevated in cats with FIP [51,52]. However, none of the above-mentioned findings are pathognomonic for FIP and should always be evaluated in conjunction with other diagnostic parameters.

#### 2.2.2. Effusion Cytology and Biochemistry

In case of effusive FIP, the fluid obtained by imaging-guided aspiration of the effusion typically exhibits viscous consistency, yellowish color and clear to turbid transparency. Subsequent biochemical and cytological analysis of the fluid are essential for the diagnostic procedure and the exclusion of other causes of effusion. Effusions associated with FIP are characterized by high protein levels (>35 g/L) and generally low total cell count (<5 × 10^9^ cells/L) [2]. However, the effusion may sometimes show increased cell counts, especially when secondary bacterial infections are involved, and more rarely, chylous appearance [53]. Cytological evaluation of a FIP effusion usually reveals a pyogranulomatous inflammatory reaction with macrophages, non-degenerated neutrophiles and a few lymphocytes [30]. The background often has eosinophilic protein-rich material. Consistent to the biochemical findings, the A:G ratio in the fluid is generally decreased (<0.4) in cases of FIP [54].

#### 2.2.3. Rivalta’s Test

The Rivalta’s test is an inexpensive and rapid method that can be easily applied to differentiate transudate from exudate effusions and is recommended for the diagnostic procedure of effusions [30]. The Rivalta’s test is not FIP-specific; however, a negative result has a high negative predictive value of 93% and helps excluding FIP from the diagnosis [32]. If the test turns out positive, further testing is required for confirmation. Rivalta’s method is easy to apply and involves the addition of an effusion’s drop to an acetic solution. If the effusion is caused by FIPV, it will typically contain high levels of proteins and inflammatory mediators which will promote precipitation of the drop leading to a positive result. If the drop dissolves in the solution, the test is considered negative, and it is suggested to rule out FIP. However, FIP diagnosis and its potential exclusion should not be based on Rivalta’s test alone, as the interpretation of the results is challenging and depends on the clinician’s experience [55].

#### 2.2.4. FNA Cytological and Fluid Analyses of CSF, and Aqueous Humor

Fine-needle aspiration (FNA) cytology, cerebrospinal fluid (CSF) analysis and aqueous humor evaluation can provide supportive information for FIP diagnosis, particularly in cases presenting characteristic clinical signs without detectable effusions. FNA of affected organs (lymph nodes, liver, and kidneys) typically reveals pyogranulomatous inflammation [56]. In cats with neurological FIP, CFS analysis is normal or characterized by high protein levels and cytology reveals neutrophilic, mononuclear or pyogranulomatous pleocytosis [34]. Comparable cytological alterations are also observed in the aqueous humour of cats with FIP associated uveitis.

### 2.3. Diagnostic Imaging

Conventional imaging techniques such as ultrasound and radiography are used to detect effusions and organ-specific abnormalities, but they do not provide any specific findings for FIP. The use of these techniques is indicated to optimize and guide tissue and effusions sampling for subsequent laboratory examinations. In neurological FIP, magnetic resonance imaging (MRI) can provide useful information. Brain MRI findings in cats with FIP include obstructive hydrocephalus, syringomyelia, foramen magnum herniation and the prominent meningeal and periventricular contrast enhancement [29,57].

### 2.4. FCoV Detection

#### 2.4.1. FCoV Antigen Immunostaining

Different immunostaining techniques can be used to detect FCoV antigen within the host cells or tissues.

Currently, the combined assessment of histopathological changes in affected tissues and the detection of FCoV antigen in macrophages by immunohistochemistry (IHC) is considered the gold standard for FIP diagnosis [2,29,30,36]. However, IHC is more feasible for post-mortem diagnosis because tissue sampling requires invasive procedures that may be unjustified in cats with a poor prognosis for FIP [58]. When using IHC for ante mortem diagnosis, it is recommended to perform tissue sampling using minimally invasive methods, such as Tru-cut biopsy (TCB) to minimize the associated risks [56]. IHC detection of FCoV antigen is considered a highly specific and reliable method for FIP diagnosis, if it is properly performed.

Similarly, cytology analysis combined with immunostaining techniques, including immunocytochemistry (ICC) and immunofluorescence (IF), performed on samples from effusions, FNAs, CSF and aqueous humor, can be highly supportive for FIP diagnosis [35,59]. Immunostaining for FCoV in effusion presents high diagnostic reliability, demonstrating up to 100% specificity [60].

Both histopathology and cytology typically reveal pyogranulomatous inflammation, while the concurrent detection of FCoV antigen by immunostaining provides confirmatory evidence for FIP. A negative immunostaining result does not exclude FCoV from the differential diagnosis, as the distribution of viral agent can vary within tissues [61].

#### 2.4.2. Molecular Detection of FCoV

Detection of FCoV RNA in blood, effusions, tissues, FNAs, CSF and aqueous humor can be achieved using reverse-transcription PCR (RT-PCR) assays, although the interpretation of the results is challenging and not necessarily confirmatory for FIP [36,62]. Firstly, detection of FCoV RNA is not FIP specific, as FCoV can also be present in cats with systemic FECV infection. Nevertheless, high FCoV viral loads in feline samples obtained from cats with clinical signs and other laboratory findings consistent with FIP can be suggestive for the diagnosis and helpful especially in non-effusive forms of the disease [63,64]. Thus, quantitative RT-PCR (RT-qPCR) assays are recommended to determine the viral loads. Moreover, due to the high mutating rate of FCoV, sequence variations in the targeted regions may compromise the RT-PCR sensitivity and lead to inaccurate results [65]. Another diagnostic approach involves the detection and characterization of FCoV S gene mutations using molecular techniques, such as sequencing or mutations-specific PCR assays [59,66]. These techniques may improve the specificity, but they do not substantially increase diagnostic accuracy and are not recommended for FIP routine diagnostic use [67]. Finally, FCoV RNA detection in fecal samples is not suggested for diagnostic purposes as it mainly indicates viral shedding rather than systemic infection, but it may be useful in monitoring FCoV circulation in multi-cat households.

#### 2.4.3. Indirect FCoV Detection

Indirect detection of FCoV relies on the measurement of anti-FCoV antibodies in the serum of FIP suspected cats by enzyme-linked immunosorbent assay (ELISA), indirect immunofluorescence antibody (IFA) or rapid immunomigration tests [68]. However, the presence of anti-FCoV antibodies only reveals previous exposure to the virus and does not specifically indicate exposure to the virulent FIPV type [30]. Consequently, the identification of anti-FCoV antibody is not considered a definitive diagnostic tool for FIP. Moreover, a negative result for serum anti-FCoV antibody does not exclude FIP from the diagnosis, as reported in a previous study [60]. Anti-FCoV antibody testing can also be performed on effusion samples or CSF, although presenting a variation in the results that ideally should be evaluated in combination with a molecular detection of the virus [69]. Interestingly, recent evidence suggests that an IFA assay targeting the FCoV nucleocapsid (N) protein combined with FCoV RNA detection may improve diagnostic accuracy in cases of effusive FIP [70].

## 3. General Management of Cats Suspected with FIP

Beyond diagnostic procedures, additional management steps may support cats suspected to have FIP. Lymphopenia is a consistent laboratory abnormality in FIP and contributes to an increase cat susceptibility to secondary infections that may accelerate the disease progression [42]. For this reason, routine infection-control precautions should be implemented when handling immunosuppressed cats with FIP. As previously discussed, FIPV is believed to arise from an internal mutation of FECV within the infected host. Consequently, even if FECV is readily transmitted between cats via the fecal-oral route, FIPV has a low transmission potential, and the isolation of a cat suspected with FIP is generally not required. Similarly, a cat with suspected FIP can usually return safely to a multi-cat household as the other cats will already have been exposed to the same FCoV strain [2]. However, a recent FIP outbreak in Cyprus, caused by the FCoV-23 strain, has raised questions about whether FIPV emergence is only the result of internal cell-tropism mutation or whether direct cat-to-cat transmission may also occur [71]. Moreover, given that FCoV can persist in the environment for extended periods, households with a history of FIP should maintain strict hygiene practices, avoid overcrowding, and closely monitor cats for any disease progression. In case of FIP-related death or euthanasia, the introduction of new cats into the household is not recommended for at least two months to minimize the risk of FCoV infection [29].

## 4. Treatment Approaches

Recent research advancements have introduced therapeutic strategies for FIP that were previously unavailable. Although these treatments are not yet fully curative, they have improved the clinical management and outcomes of affected cats. Table 2 summarizes the major therapeutic approaches, which are discussed in detail in this section.

### 4.1. Nucleoside Analogs

Nucleoside analogs (NAs) have long been used as therapeutic agents against different viruses, including CoVs. Upon activation by cellular kinases, NAs are recognized by the viral polymerase and used as nucleotide substrates into the nascent viral strand, resulting in premature chain termination or lethal mutagenesis, thereby inhibiting viral replication [72].

The adenosine analog GS-441524, previously used as antiviral against different human viruses, was proposed in 2018 as a potential treatment for FIP [73], fundamentally changing the disease’s prognosis. Since then, several research studies, including in vitro assays, experimental infections and field clinical trials, have assessed its antiviral activity and reported excellent results with success rates of 83% when administered alone and up to 90% in combination with other antivirals [74,75]. Briefly, GS-441524 can be administered either subcutaneously (SC) or orally (PO), with higher doses recommended for neurological or ocular FIP, as well as for retreatment in case of disease recurrence [76,77]. Although most studies have investigated an 84-day treatment protocol, shorter treatment may also be effective [78]. Some minor adverse effects due to GS-441524 include lymphocytosis, eosinophilia, increased alanine aminotransferase (ALT) levels and nephrolithiasis [79,80]. Despite its proven efficacy, GS-441524 remains unlicensed and is not commercially available worldwide, although it is allowed in some countries as an extemporaneously prepared and highly expensive product.

Remdesivir (RDV) or GS-5734 is a nucleoside analog prodrug that is converted in vivo to GS-441524, sharing the same antiviral mechanism. Originally, it was designed for the Ebola virus, and later, it was repurposed for the treatment of SARS-CoV-2 infection [81]. RDV is currently authorized for human use in many countries, while in veterinary medicine, only compounded drugs are available. A limitation of RDV therapy is the painful administration and high cost, as it is usually injected intravenously (IV) or SC. Although RDV was initially considered to have low oral bioavailability, recent studies have reported 79–86% efficacy in treating FIP following oral administration [82,83]. RDV is mainly used for FIP treatment when GS-441524 is not available, although combination protocols involving both compounds have yielded promising results [79,84,85].

Molnupiravir (MPV) is an orally available nucleoside analog prodrug with broad spectrum antiviral activity. Authorized for COVID-19 treatment since 2021, it was first used off-label for FIP in 2022 [86,87]. Subsequent studies have demonstrated that MPV is similarly effective and safe as GS-441524, while it has shown favorable outcomes as a first-line, maintenance or rescue treatment in case of other antivirals’ failure [88]. The reported adverse effects of MPV include neutropenia, nausea, dysrexia and elevated liver enzyme levels [89,90,91]. MPV may therefore represent a more affordable and accessible alternative for FIP therapy in countries where veterinarians are permitted to prescribe human medicine.

Other nucleoside analogs, including mefloquine, ribavirin, 6-azauridine, 3-deaguanosine and the purine analog adenine arabinoside, have also been evaluated for FIP treatment. However, these drugs have demonstrated lower antiviral efficacy or higher cytotoxicity [92].

### 4.2. Viral Protease Inhibitors

The 3-chymotrypsin-like protease (3CLpro) is a highly conserved enzyme among CoVs and plays a crucial role for viral replication by cleaving the viral polyprotein into functional NSPs. Accordingly, 3CLpro has become a target for antiviral drug development in both human and veterinary medicine.

Peptidyl compounds such as GC376 and its active form GC373 have been shown to inhibit the 3CLpro of FIPV, thereby suppressing viral replication [93]. GC376 has demonstrated efficacy, both in vitro and in vivo, in experimentally infected cats as well naturally occurring cases of FIP [94,95]. Moreover, it has shown antiviral activity against SARS-CoV-2 [96]. Combination therapy of GC376 and GS-441524 has been associated with improved efficacy and reduced treatment duration [97]. The reported adverse effects are generally mild, however further research and clinical trials are warranted [29]. Currently, GC373/GC376 are not commercially available for human or veterinary use.

### 4.3. Interferons

Interferons (INFs), key proteins of the innate antiviral immune response, trigger the expression of specific genes, leading to the establishment of an antiviral state [98].

Recombinant feline IFN-omega (rfIFN-ω), a type I IFN commercially available for veterinary use, is employed to treat various feline viral infections [99]. However, evidence regarding its efficacy in treating FIP remains inconclusive. While Ishida et al. reported an efficacy rate of 33.3% in cats treated with rfIFN-ω, a subsequent placebo-control trial found no significant difference in FIP progression between cats receiving rfINF-ω combined with glucocorticoids and those given glucocorticoids alone [100,101]. An in vitro study demonstrated that the combination of hydroxychloroquine (HCQ), an anti-malaria drug, and rfINF-ω blocked cellular infection by FIPV type I, but showed reduced efficacy against FIPV type II, likely due to the latter’s ability to inhibit the expression of IFN type I [99,102]. Despite variable outcomes, rfINF-ω has been suggested as a combination treatment with other antivirals or as a maintenance treatment in cats with FIP, based on reports of its potential to enhance antiviral activity [76].

### 4.4. Polyprenyl Immunostimulant

Polyprenyl Immunostimulant (PI) is an orally administered immunomodulatory agent, licensed for the management of feline herpesvirus (FHV) [103]. PI enhances cell-mediated immunity by upregulating the innate immune response via Toll-like receptors. Although PI cannot be considered a curative therapy for FIP, previous investigations have associated its use with prolonged survival especially in non-effusive FIP cases, while combination with glucocorticoids should be avoided [104,105]. A retrospective analysis reported a survival time of approximately eight years in cats with FIP who received PI for at least one year [106]. More recently, a proof-of-principal study suggested that combining PI with MPV can limit the treatment duration in immunocompromised cats with FIP [107]. Further research is required to define PI’s role in FIP therapy and to clarify its synergism with other antiviral drugs.

### 4.5. Other Compounds

Research and development of other potential antiviral agents for FIP are ongoing, although in vivo studies are still required to confirm their therapeutic potential and efficacy. Flavonoids, and more specifically isoginkgetin and luteolin, have demonstrated inhibitory activity against FIPV infection in Crandell-Rees Feline Kidney (CRFK) cells in vitro. Isoginkgetin appears to interfere with early intracellular viral replication, while luteolin acts at a post-viral entry stage of infection [108]. A novel molecule, K31, previously used against hantaviruses, targets the N protein and has been reported to inhibit FCoV replication in cell culture [109]. Moreover, a non-nucleoside inhibitor of the RNA-dependent RNA polymerase, ERDRP-0516, suppressed FIPV type II infection in CRFK cells in a dose-dependent manner [110]. Other compounds, including curcuminoids, Thymus vulgaris essential oil, Vigna Radiata extract, and α-mangostin, have been reported to have antiviral effects against FCoV in vitro [111,112,113,114].

## 5. Prevention

### 5.1. Vaccination

Currently, an intranasal vaccine for FIP (Felocell FIP, Zoetis, Parsippany, NJ, USA) is commercially available in the USA and several European countries [115]. However, it is not recommended by the European Advisory Board on Cat Diseases (ABCD) as its efficacy remains questionable [116]. This vaccine was initially developed in 1991 using a temperature-sensitive mutant of FCoV type II strain DF2 [117,118]. Its primary objective was to elicit a strong mucosal immune response by inducing IgA production and activating cell-mediated immunity [119]. Given that FCoV type II is relatively rare in the field, the vaccine is unlikely to provide effective protection in the general cat population. Moreover, administration is recommended in kittens older than 16 weeks of age to prevent FCoV infection, but many kittens are already exposed to the virus at this age, especially in multi-cat environments.

The S protein is usually the primary target for vaccine development against CoVs. Chawla et al. (2023) proposed a vaccine candidate for FIP that targets multiple epitopes within the S gene using immunoinformatic approaches [120]. However, in the case of FIPV, S protein-based vaccines can lead to antibody-dependent enhancement (ADE) of the infection. Experimental studies have demonstrated that non-neutralizing antibodies bound to the viral surface can facilitate viral entry to monocytes or macrophages, which are the main cells supporting FIPV replication, resulting in increasing viral spread and disease [121]. This seems to be a major challenge for S protein-focused FIPV vaccine strategies. To address this issue, Takano et al. (2014) suggested the development of peptide-based vaccines targeting T helper 1 (Th1) epitopes, while avoiding ADE-associated epitopes [122]. Another study developed a recombinant vaccine using Bacillus subtilis spores fused to a peptide derived from the heptad repeat 2 (HR2) domain of a serotype II FECV strain [123]. Although administration of this vaccine in a mouse model triggered an immune response against the virus, its efficacy appears to be limited only against the less common serotype II.

Several vaccine development efforts have focused on the FIPV N protein, as it is highly genetically conserved in both serotypes [124,125]. An experimental infection study, in which cats were immunized with a recombinant type I FIPV vaccine expressed by the N-gene baculovirus, reported no induction of neutralizing antibodies but demonstrated antigen-specific cell-mediated immunity, with a 75% survival rate after FIPV challenge [126]. Another study introduced a recombinant adenovirus vaccine, administered IM, expressing the FCoV N protein, and evaluated its efficacy in mice and cats that reported stimulation of cellular immunity [127]. More recently, a lipid nanoparticle-encapsulated mRNA vaccine targeting the N protein has been developed, with in vivo trials in mice eliciting both humoral and cellular immune responses [128].

### 5.2. Measures to Reduce the Risk of FCoV Infection

Limiting the risk of FCoV is crucial, as infection represents the first step that may lead to the development of FIP. Consequently, prevention strategies focus on both reducing FCoV transmission and controlling viral circulation when infection is already present [129]. As discussed in Section 3, household-level measures to reduce the risk of FCoV include good hygiene practices and avoiding overcrowding. Specifically, households should provide an adequate number of litter trays relative to the number of cats, ensure frequent cleaning and closely monitor cats for clinical signs of disease.

Breeding catteries represent a high-risk environment for FCoV transmission due to high population density and frequent animal movement. Therefore, strict hygiene protocols, effective infectious disease control measures and stress reduction strategies are essential. Cats should be monitored using RT-qPCR for FCoV detection, before entry into a cattery, at regular intervals during their stay and before leaving [130]. Screening should include also other viral pathogens. Some studies suggest isolating animals identified as viral shedders to limit transmission, however this approach is challenging, as viral shedding may persist for months and repeated sampling is required [31]. Similar precautions should be applied for newborn kittens. They should be housed in a clean environment with minimal exposure to FCoV, particularly during the first weeks of life when susceptibility is highest. Isolation of kittens from mothers when the latter are viral shedders has been proposed, but remains controversial and not always recommended [131]. Complete eradication of FCoV from a cattery is difficult, especially once the virus has already been established. Nevertheless, the implementation of appropriate preventive measures can significantly reduce viral load, limit transmission and decrease the risk of FIP development.

## 6. Conclusions

Ultimately, advances in the understanding and management of FIP have dramatically changed the prognosis and expectations associated with the disease. Nevertheless, establishing a definitive ante-mortem diagnosis remains a major challenge. Confirmation of the presence of FIPV, the virulent mutant form of FCoV, relies on its detection within lesions by IHC, a diagnostic approach rarely feasible in living FIP patients. Consequently, veterinarians rely on combinations of less invasive tests and procedures which often produce nonspecific results for FIP. Although high FCoV RNA concentrations should be a warning sign and raise FIP suspicion, this parameter also lacks specificity for the disease. Therefore, personalized interpretation of clinical signs and laboratory abnormalities, with repeated examinations as the disease evolves, is recommended. Diagnostic algorithms tailored to the different forms of FIP may support veterinarians in this process [2].

FIP is no longer the invariably fatal disease it was once considered to be. Although current therapeutic options are promising rather than curative, they have significantly improved FIP remission and survival rates. Among these, nucleoside analogs represent the most significant recent development in the treatment of FIP. Importantly, a regulatory shift is emerging in Europe towards the acceptance of these antivirals. Among the latest regulatory measures, the Italian Ministry of Health has authorized the off-label use of Remdesivir (as Veklury) and of its active metabolite for GS-441524 for FIP treatment in the absence of veterinary medicinal products [132]. This decision allows veterinary prescription under professional responsibility and may support the adoption of similar policies in more countries. Continued research into these agents, along with efforts to enhance their accessibility, as well as the exploration of additional antiviral strategies, is crucial. At the same time, the lack of an available effective vaccine has already prompted research efforts for vaccine development. Recent mRNA-based approaches may prove to be promising vaccine strategies for the future prevention of FIP, especially given the widespread use of mRNA vaccines against HCoVs [128,129]. Overall, FIP prognosis will remain poor without appropriate therapy and management. In this context, owner counseling is essential to support informed decision-making focused on treatment options and quality of life, with euthanasia considered only when treatment is not feasible or fails to achieve an acceptable welfare outcome. Further research and coordinated action across veterinary and pharmaceutical fields are required.

## Figures and Tables

**Table 1:** Principal diagnostic methods and relative clinical utility.

Sample Type	Diagnostic Method	Clinical Utility	Reference
Blood	Hematology Serum biochemistry	Supportive but non-specific findings. Low–moderate sensitivity and low specificity.	[29]
	Molecular detection (RT-PCR, RT-qPCR)	FCoV can also be present in cats with systemic FECV infection. Very high titers may raise FIP suspicion. Moderate sensitivity and low specificity.	[30]
	Antibody detection (ELISA, IF)	Anti-FCoV antibodies can be detected in cats without FIP. If negative, FIP is unlikely. High sensitivity and very low specificity.	[31]
Effusion	Cytology	Typically, pyogranulomatous inflammatory reaction with macrophages. Moderate to high sensitivity and moderate specificity.	[2]
	Rivalta’s test	Excellent NPV. Useful to exclude FIP. High sensitivity and moderate specificity.	[32]
	Molecular detection (RT-PCR, RT-qPCR)	Strong supportive evidence in case of effusive FIP. High sensitivity and moderate to high specificity.	[33]
FNA CSF Aqueous humor	Cytology	May demonstrate pyogranulomatous inflammation with macrophages. Moderate sensitivity and specificity.	[34]
	Immunostaining (ICC, IF)	Strong confirmatory evidence of FIP. Moderate to high sensitivity and very high specificity.	[35]
	Molecular detection (RT-PCR, RT-qPCR)	Useful in combination with cytology and antigen detection. High sensitivity and moderate specificity.	[31]
Tissue	Histopathology	Moderate specificity but low sensitivity depending on the lesion.	[36]
	Immunostaining (IHC)	Diagnostic gold standard for FIP. High sensitivity and very high specificity.	
	Molecular detection (RT-PCR, RT-qPCR)	High titers are reliable for FIP confirmation in combination with other tests. High sensitivity and moderate specificity.	

Note: FIP, feline infectious peritonitis; FCoV, feline coronavirus; FECV, feline enteric coronavirus; RT-PCR, reverse transcription polymerase chain reaction; RT-qPCR, quantitative reverse transcription polymerase chain reaction; ELISA, enzyme-linked immunosorbent assay; IHC, immunohistochemistry; ICC, immunocytochemistry; IF, immunofluorescence; FNA, fine-needle aspirate; CSF, cerebrospinal fluid; and NPV, negative predictive value. Diagnostic sensitivity, specificity, and predictive values are expressed using a qualitative scale (low, moderate, high, very high) based on the ranges and consistency of results reported in the literature, rather than pooled numerical estimates, due to inter-study heterogeneity in the methodology and case definition.

**Table 2:** Treatment options for FIP, categorized by therapeutic class and brief comments for each agent.

Therapeutic Class	Agent	Comments/Notes
Nucleoside Analogs	GS-441524	Promising clinical results; reported success rates ~83% as monotherapy and up to ~90% in combination therapy; not licensed and available only as costly extemporaneous preparations
	Remdesivir (GS-5734)	Prodrug of GS-441524; promising results as monotherapy or in combination protocols; not approved for veterinary use; available only as extemporaneous formulations
	Molnupiravir	Promising results; generally, more affordable than GS-441524-based treatments; approved for human use and potentially accessible under the prescribing cascade
Viral Protease Inhibitors	GC376/GC373	Good antiviral activity; particularly promising in combination therapy; not commercially available
Interferons	rfINF-ω	Suggested as adjunct or maintenance therapy rather than primary treatment; commercially available for veterinary use
Immunomodulators	PI	May help reduce treatment duration when used in combination therapy; avoid co-administration with glucocorticoids
Non-nucleoside Inhibitors	ERDRP-0516	Preliminary antiviral activity reported in vitro only
Natural Compounds	Flavonoids	Antiviral activity reported in vitro only
	K31	Preliminary antiviral activity reported in vitro only
	Curcuminoids	Immunomodulatory and antiviral effects reported in vitro only
	*Thymus vulgaris* essential oil	Antiviral activity reported in vitro only
	*Vigna Radiata* extract	Preliminary antiviral activity reported in vitro only
	α-mangostin	Antiviral activity reported in vitro only

## Data Availability

No new data were created or analyzed in this study. Data sharing is not applicable to this article.
